# Clonal Dynamics After Allogeneic Hematopoietic Stem Cell Transplantation from Related Donors: A Case Series

**DOI:** 10.3390/ijms27188008

**Published:** 2026-09-09

**Authors:** Valentina Giudice, Denise Morini, Maddalena Langella, Francesco Verdesca, Francesca Velino, Anna Maria Sessa, Simona Caruso, Martina De Leucio, Pasqualina Scala, Italia Conversano, Anna Maria Della Corte, Danilo De Novellis, Bianca Serio

**Affiliations:** 1 Hematology and Transplant Center, University Hospital “San Giovanni di Dio e Ruggi d’Aragona”, 84131 Salerno, Italy; 2 Department of Medicine, Surgery and Dentistry “Scuola Medica Salernitana”, University of Salerno, 84081 Baronissi, Italy

**Keywords:** clonal hematopoiesis, clonal dynamics, hematopoietic stem cell transplantation, matched-related donor

## Abstract

Allogeneic hematopoietic stem cell transplantation (HSCT) serves as a critical model for investigating clonal dynamics, as hematopoietic reconstitution occurs amidst significant proliferative stress and immune-mediated pressures. This study utilized longitudinal next-generation sequencing of a 30-gene panel to track clonal evolution in 20 patients and their matched-related or haploidentical donors. The findings revealed that post-transplant hematopoiesis is frequently driven by donor-derived clones, with *DNMT3A* being the most prevalent mutation in the study population. A key finding was the significant association between the presence of the donor *TET2* Leu1721Trp variant and reduced overall survival in recipients (median 14.3 months compared with 50.8 months for wild type; *p* = 0.0100). Additionally, the study showed that the re-emergence of recipient-derived clones, particularly oncogenic *DNMT3A* mutations, frequently preceded graft failure or disease relapse. These findings suggest that, although donor clonal hematopoiesis is generally considered clinically neutral, specific genetic variants can profoundly influence clinical outcomes, suggesting that comprehensive molecular screening of donors and longitudinal molecular monitoring of recipients may be essential to optimize transplant outcomes and personalize post-HSCT management.

## 1. Introduction

Clonal evolution refers to the selection of specific malignant cell populations under continuous selective pressures, such as immune surveillance or therapeutic interventions, whereas clonal hematopoiesis (CH), commonly observed with aging, is characterized by the presence of hematopoietic clones carrying mutations in cancer-associated genes without necessarily being associated with overt hematologic malignancy [1,2,3]. Somatic mutations accumulate physiologically throughout life, mainly as single-nucleotide variants, insertions/deletions (indels), or chromosomal abnormalities, leading to genetically distinct clonal populations and reduced stem cell diversity [4,5]. Hematopoietic stem cells (HSCs) acquire approximately 20 somatic mutations annually, with those persisting and occasionally conferring a selective advantage, driving clonal expansion, particularly during aging. This process has been proposed as a molecular clock for both normal and premalignant hematopoiesis [6,7,8]. The development of CH and clonal behavior largely depend on the underlying genotype, with clonal dynamics and fitness varying according to the biological context [9,10,11,12]. With the widespread implementation of next-generation sequencing (NGS) in routine clinical practice, clonal alterations are increasingly detected, even in individuals without clinical or laboratory evidence of hematological malignancies, defining the condition known as clonal hematopoiesis of indeterminate potential (CHIP) [13,14]. CH and CHIP are closely associated, as the first is a broader term identifying the presence of a potential pathogenic clone within normal hematopoiesis, while the latter is a clinically well-defined condition, where somatic mutations in leukemia-associated genes (frequently *DNMT3A*, *TET2*, and *ASXL1*) have a variant allele frequency (VAF) ≥ 2% (≥4% for X-linked genes in males), in the absence of marrow dysplasia, and peripheral blood (PB) cytopenia(s) [15]. Although initially considered of uncertain clinical significance, individuals with CHIP have an annual risk of progression to acute myeloid leukemia (AML) estimated at <1%, and also have an increased risk of cardiovascular events and cardiovascular mortality [16].

Hematopoietic stem cell transplantation (HSCT) remains the only curative therapeutic option for certain hematological malignancies, such as AML, as this procedure aims at repopulating the bone marrow (BM) of patients with a stem cell compartment obtained from a healthy donor after conditioning regimens using high-dose chemotherapies [17]. Allogeneic HSCT uniquely reconstitutes hematopoiesis by introducing a genetically distinct immune system, resulting in new immuno-hematological interactions between donor cells and the recipient microenvironment [18]. This procedure offers a model for studying clonal dynamics, as transplanted cells expand and adapt to pathological conditions shaped by immunological, inflammatory, and pharmacological factors. Moreover, selective pressures mainly determined by the proliferative stress for the BM reconstitution may favor the expansion of certain clones, based on their fitness [3,12,19,20]. Indeed, early post-HSCT reconstitution can be transiently oligoclonal and influenced by donor mutations or surviving recipient subclones [21]. Thus, post-transplant BM reconstitution is a dynamic process, involving clonal competition and selection and niche remodeling, and molecular profiles of donor cells, especially the presence of CHIP, may affect stem cell expansion [19]. CH may significantly impact BM reconstitution and the patient’s clinical outcomes, as the donor’s CHIP may result in cytopenias and myelodysplastic syndromes (MDS) in recipients [21,22,23]. In addition, conditioning regimen-induced myelotoxicity and immunosuppression can foster new genetic alterations or affect residual stem cells; therefore, post-transplant emerging CH may independently arise from the original disease or overlap with it, thus influencing outcomes [24]. NGS improves molecular characterization over traditional monitoring by identifying low-frequency somatic mutations and distinguishing between disease recurrence, donor-derived clones, and new mutations. Longitudinal sequencing helps assess patient and donor clonal structures, predict transmission risks, track disease relapses versus new clonal emergence, and stratify long-term risk [25]. Integrating clinical and molecular data is crucial for understanding post-transplant hematopoiesis and detecting detrimental clonal changes [26]. Based on these considerations, we aimed to characterize clonal dynamics in donors and recipients before and after allogeneic match-related or haploidentical HSCT by longitudinal NGS, to clarify the biological and prognostic significance of CH and clonal sweep in transplant recipients.

## 2. Results

### 2.1. Molecular Landscape and Clonal Dynamics of Patients

**Figure 1:**
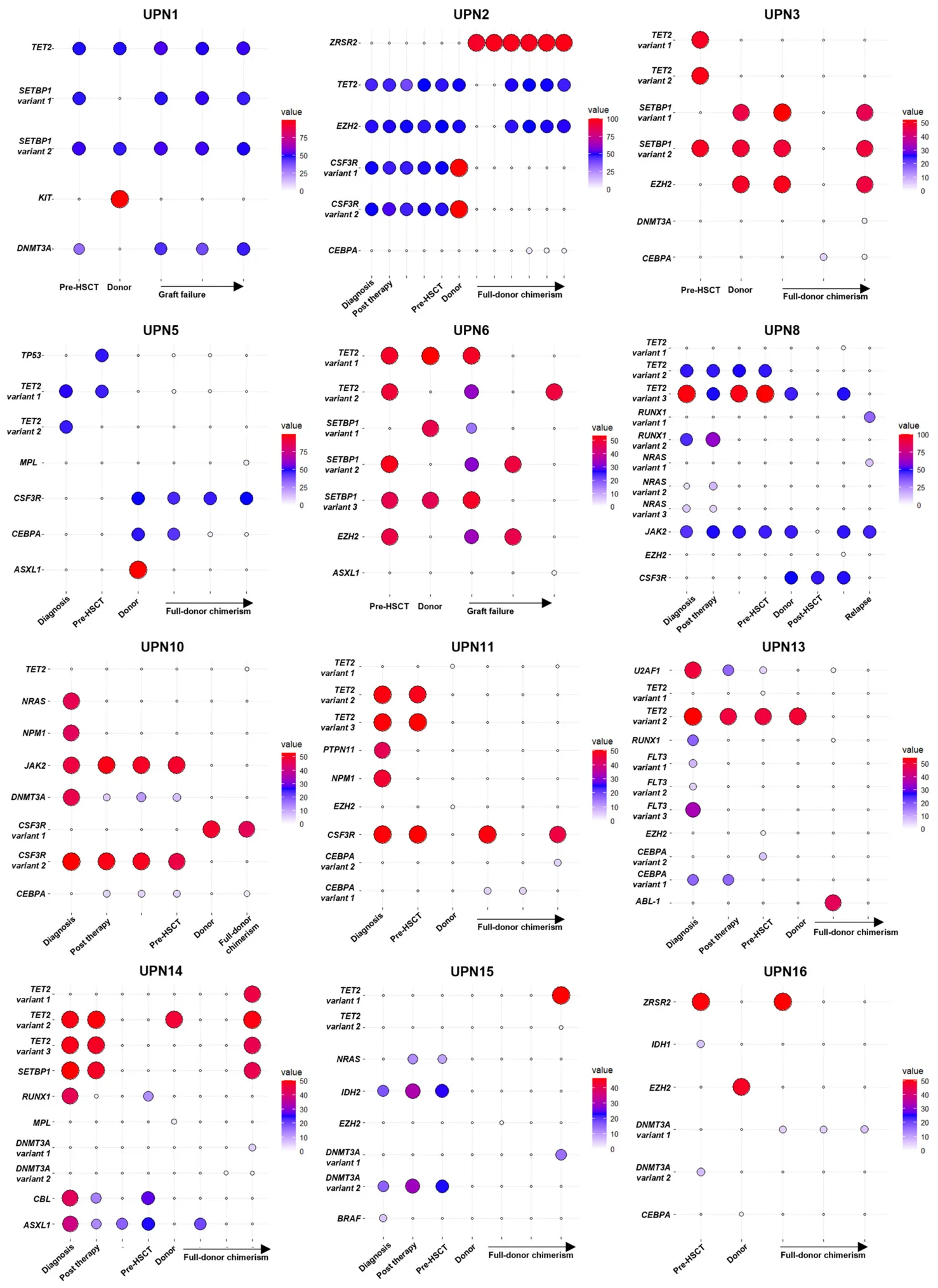
Mutational landscapes before and after matched-related donor transplantation. Balloon plots showing the clonal dynamics of representative patients before and after transplant (HSCT), according to the outcomes. The size of each dot is proportional to the variant allele frequency (VAF) value, and color gradient ranges from white (VAF 0%) to red (VAF 100%).

To characterize clonal dynamics in transplant recipients, molecular landscapes of recipients before and after HSCT and of their corresponding donors were longitudinally analyzed to identify clonal trends in our case series of patients receiving a diagnosis of AML and treatments according to current guidelines (Figure 1 and Supplementary Table S1) [26,27,28,29,30,31,32,33,34,35]. For example, UPN1 received haploidentical transplantation from his sister and experienced a graft failure. The patient had an oncogenic mutation in *DNMT3A*, absent in the haploidentical donor, who carried a VUS in c-*KIT*. After the graft failure, the patient showed re-emergence of the oncogenic *DNMT3A* mutation, suggesting loss of donor-derived engraftment and restoration of recipient-derived hematopoiesis.

UPN8 underwent haploidentical transplantation from his son. At diagnosis, he had driver mutations in *NRAS* Gly13Arg (VAF, 12%) and Gly13Asp (VAF, 7.3%), which persisted during induction therapy (VAF 9.7% and 15.5%, respectively). Moreover, one VUS in *JAK2*, two in *TET2*, and one in *RUNX1* were documented. Before transplant, no oncogenic mutations were observed. The donor had a non-neoplastic mutational landscape, with a VUS in *CSF3R*, and another two variants shared with his father in *JAK2* and *TET2*. In the post-transplant follow-up, a donor-derived hematopoietic reconstitution was initially observed; however, the patient had disease relapse and his mutational profile displayed a clonal sweep, although the *NRAS* and *RUNX1* genes again mutated, while in different exons (Gln61Lys, VAF 13.5%; and Lys83Gln, VAF 33.5%). Most interestingly, after transplant, the patient showed a variant in *TET2* carried by the donor and present before transplant, and also a new one (Gln1548del, 3.2%). However, both alterations disappeared at the time of disease relapse.

UPN10 received an MR donor transplantation from his sister. The patient showed persistence of the oncogenic mutation *JAK2* Arg564Leu (VAF, 48.1%) from diagnosis to induction, although in CR before HSCT. His sister only had a VUS in *CSF3R*. Surprisingly, after transplant, the patient lost the oncogenic *JAK2* mutation, and acquired his sister’s *CSF3R* alteration, confirming a donor-derived hematopoietic reconstitution. A similar dynamic was observed for UPN11, who underwent allogeneic HSCT from her MR sister.

UPN14 underwent allogeneic transplantation from his brother. Molecular analysis in the pre-transplant phases showed the presence of a complex mutational structure associated with the underlying disease, with partial persistence of the alterations after induction therapy. The donor had a molecular profile compatible with non-neoplastic CH, with a VUS in the *MPL* and *TET2* genes. In post-transplant follow-up, we observed the disappearance of the pre-transplant alterations, associated with donor-derived hematopoietic reconstitution. However, a clonal dynamic characterized by the reappearance (*SETBP1*) and occurrence of novel oncogenic (*DNMT3A*, Arg891Profs*30, VAF 1.24%) molecular alterations was documented, indicating ongoing post-transplant clonal remodeling with a CH-driven BM reconstitution. A similar dynamic was observed for UPN15, who received a haploidentical transplantation from her sister, and for UPN16, who had an MR donor allogeneic HSCT.

Finally, five recipients (UPN9, 17, 18, 19, and 20) underwent allogenic HSCT from donors without detectable oncogenic mutations or VUS. Two remain alive in CR, whereas three died because of disease progression (UPN18), acute graft-versus-host disease (UPN20), or infectious complications (UPN17).

### 2.2. Frequency and Distribution of Mutations

**Figure 2:**
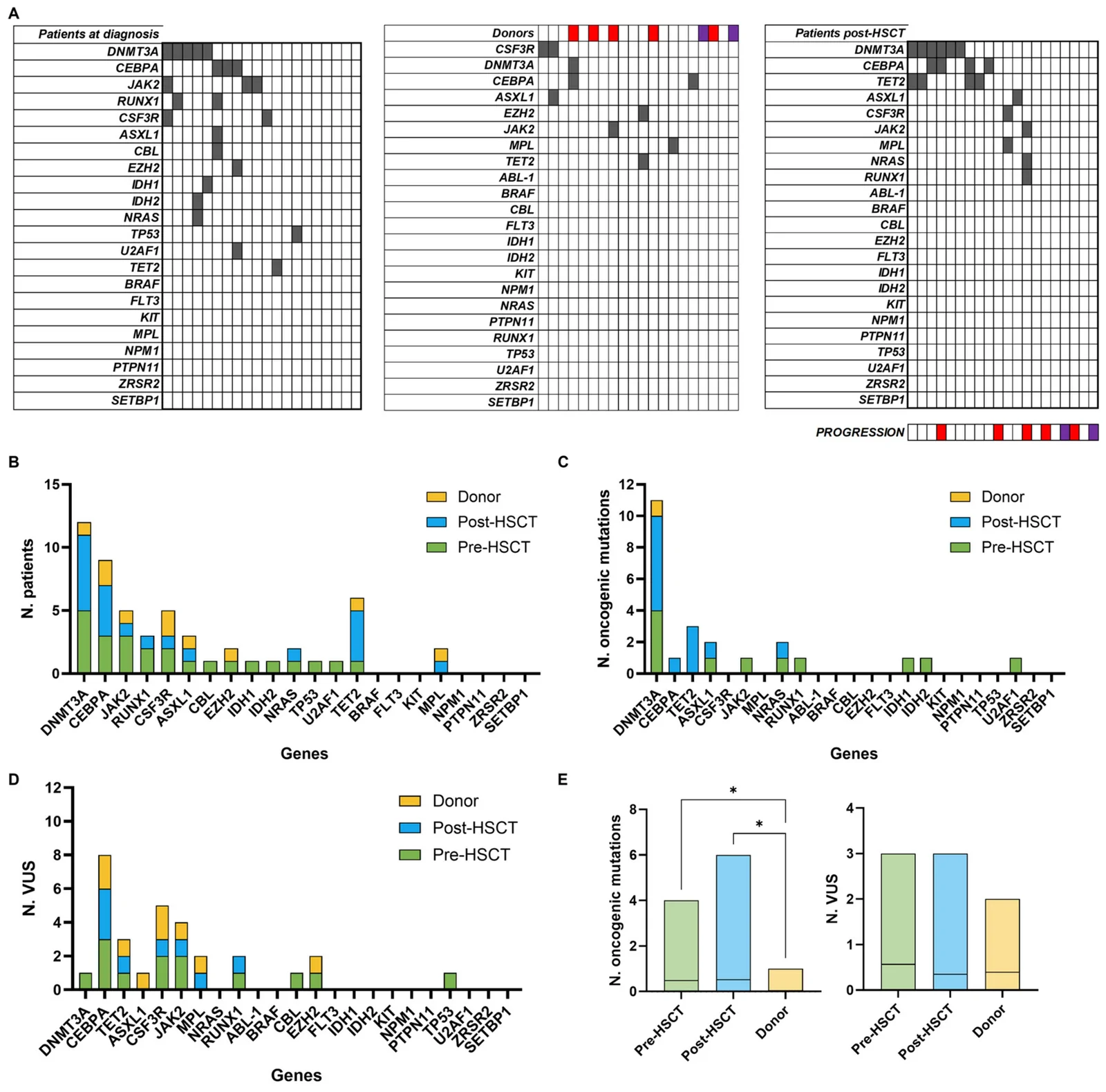
Frequency and distribution of variants in patients and donors. (**A**) Molecular landscapes are displayed for patients at diagnosis (**left**) or after hematopoietic stem cell transplantation (**right**), and for related donors (**central**). Alterations are shown in gray. Clinical outcomes for patients are represented for each donor (upper raw on the central panel) and recipient (lower raw on the right panel), showing deaths for disease relapse in red or for other causes (graft-versus-host disease or infectious complication) in purple. (**B**) The number of patients before or after HSCT and donors carrying oncogenic mutations or variants of unknown significance (VUS) is reported, as well as (**C**) oncogenic mutations or (**D**) VUS, respectively. (**E**) The total number of oncogenic mutations (**left**) or VUS (**right**) in patients before and after transplant and matching donors was compared between groups by two-way analysis of variance with uncorrected Fisher’s LSD for multiple comparisons. *, *p* < 0.05.

Subsequently, the mutational landscape was visualized for pre- and post-transplant patients and separately for donors, to highlight the distribution of recurrently altered genes (Figure 2).

In the pre-transplant setting, *DNMT3A*, *CEBPA*, *JAK2*, *RUNX1*, and *CSF3R* were the most frequently mutated genes (including both oncogenic mutations and VUS), while *ASXL1*, *CBL*, *EZH2*, *IDH1*/*2*, *NRAS*, *TP53*, *U2AF1*, and *TET2* were less frequently mutated. No alterations were observed in *BRAF*, *KIT*, *MPL*, *NPM1*, *PTP11*, *ZRSR2*, and *SETBP1* genes. After HSCT, *DNMT3A* and *CEBPA* remained the most frequently mutated genes, while *TET2* displayed more alterations compared to the pre-transplant setting (one patient before transplant vs. four subjects after transplant). *ASXL1*, *CSF3R*, *JAK2*, *MPL*, *NRAS,* and *RUNX1* were found to be less frequently altered. Among the recipients, five died after transplant for disease relapse, and two of them had 2–3 clones carrying oncogenic/VUS mutations, while the other three subjects had no molecular alterations in investigated genes. In the donor setting, only one donor had a potentially oncogenic *DNMT3A* mutation, while all other observed alterations were VUS. In detail, *CSF3R*, *DNMT3A*, and *CEBPA* were the most frequently mutated genes, while *ASXL1*, *EZH2*, *JAK2*, *MPL*, and *TET2* less frequently showed alterations. The cumulative distribution of molecular alterations in patients and donors was analyzed, confirming *DNMT3A* (22.2%) as the most frequently altered gene in the entire cohort, followed by *CEBPA* (16.7%), *TET2* (11.1%), *JAK2* (9.3%), *CSF3R* (9.3%), *RUNX1* (5.6%), and *ASXL1* (5.6%). *DNMT3A* alterations were observed both in donors and patients before and after HSCT, while other mutations were observed only before transplant, such as those in *IDH1*/*2*. Next, molecular alterations were also differentiated based on their pathogenicity, and *DNMT3A* accounted for the largest proportion of pathogenic variants pre- and post-transplant (45.8% of total oncogenic mutations), and in one case in the donor setting. Variants in *ASXL1*, *JAK2*, *IDH1*/*2*, and *U2AF1* were observed only before transplant, while other variants, such as in *CSF3R*, were documented in the post-transplant period. We then quantified, for each patient before and after HSCT and for each donor, the burden of oncogenic alterations, and compared this between groups. In particular, the number of oncogenic alterations in patients, both before (*p* = 0.0375) and after transplantation (*p* = 0.0228), even in CR subjects, was significantly greater than in donors. Similarly, the distribution of VUS was studied, showing that these variants were more commonly observed in *CEBPA*, *CSF3R*, *JAK2*, and *TET2* genes, with VUS in *DNMT3A*, *CBL*, and *TP53* only described in the pre-transplant setting. Unlike oncogenic alterations, no significant differences in VUS distribution were observed between pre- and post-transplant recipients compared to donors (*p* = 0.1191 and *p* = 0.6930, respectively).

### 2.3. Clonal Dynamics and Clinical Outcomes

**Figure 3:**
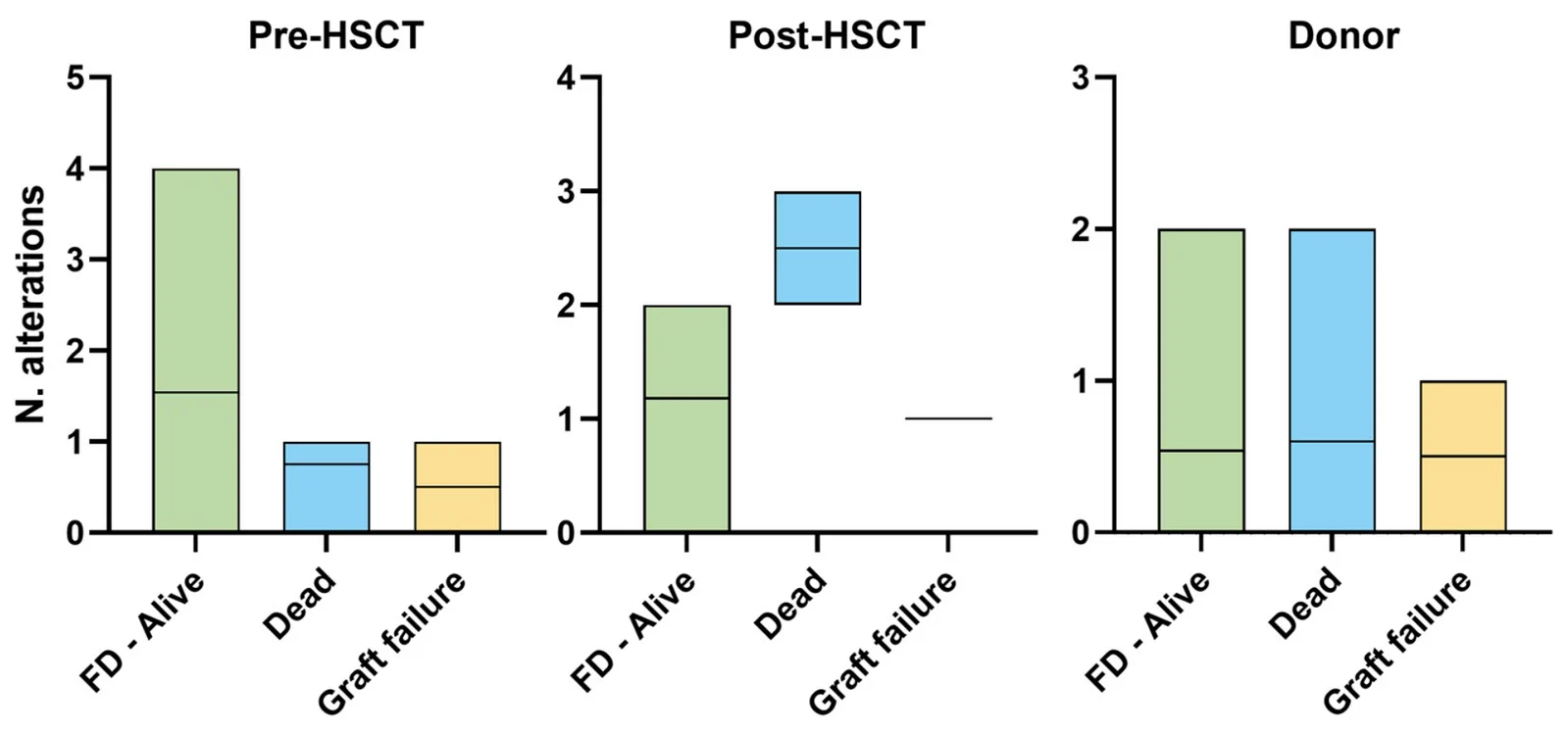
Frequency of variants based on clinical outcomes. Patients were first stratified according to their clinical outcomes: full donor chimerism (FD) and alive; dead; and those who experienced graft failure. Subsequently, the number of oncogenic mutations and variants of unknown significance was compared between groups according to the number of alterations in patients before hematopoietic stem cell transplantation (HSCT; (**left**)), after transplant (**central**), or in donors (**right**).

We next examined the number of clonal alterations, both pathogenic and VUS, for each patient, and subjects were divided according to clinical outcomes: graft failure (UPN1 and UPN6); disease progression (UPN4, UPN7, UPN8, UPN12, and UPN18); and CR and full donor chimerism (Figure 3). Although these differences did not reach statistical significance, because of the small number of patients for each group, patients with post-transplant disease recurrence showed a trend to have a higher number of molecular alterations than those in CR. Indeed, patients with disease relapse had a median of 2 alterations (range, 2–3), slightly higher than that in subjects in CR (median 1, range 0–2; *p* = 0.0724) or who had a graft failure (median 1; *p* = 0.0865). No association was observed between the number of pre-transplant recipient or donor alterations and post-transplant clinical outcome.

### 2.4. Distribution of TET2 Variants and Association with Clinical Outcomes

Based on the post-transplant appearance of *TET2* alterations, we next focused on its mutational status, and on possible associations of the Leu1721Trp variant with clinical outcomes. In our case series, patients whose donors carried this variant all died (*p* = 0.0123), although the number of subjects was still too limited and this result requires a much larger population to be confirmed. Conversely, the presence of this variant in patients before or after HSCT was not associated with clinical outcome (*p* > 0.9999).

Next, a survival analysis was conducted in the whole cohort, showing a median overall survival (OS) of 50.7 months (95%CI, 46.1-n.v.). Patients were divided according to the donor’s *TET2* mutational status (wild type [WT]; carriers of variants like Ile1762Val; or carriers of Leu1721Trp variant), and clinical outcomes were compared between groups. Although our population was limited, OS tended to be shorter in those patients whose donors were *TET2* Leu1721Trp carriers compared to WT donors or carriers of other variants (median OS, 14.3 months vs. 50.8 months vs. not reached, Leu1721Trp vs. WT vs. other variants; *p* = 0.0100). In addition, a principal component analysis was conducted including OS, outcome (alive/dead), recipient age, number of pre-transplant mutations, and number of donor molecular alterations. In detail, patients with different *TET2* mutational status tended to cluster separately, and those with the *TET2* Leu1721Trp variant tended to have a lower pre-transplant clone number compared to WT and carriers of other variants (both *p* = 0.1152), while they tended to have a higher number of clones after HSCT compared to WT (*p* = 0.0641) and carriers of other variants (*p* = 0.1157).

Finally, a preliminary multivariate analysis was conducted to explore possible risk factors that negatively affect OS (Table 1). The pre-transplant recipient’s WT *TET2* mutational status (estimate, −37.03; 95%CI, −61.31 to −12.76; *p* = 0.0079) could represent a protective factor. Given the limited sample size, these findings should be considered hypothesis-generating and warrant validation in larger prospective cohorts.

## 3. Discussion

Clonal hematopoiesis is increasingly recognized as a clinically relevant biological state associated with hematological malignancies, cardiovascular morbidity, and inflammatory complications [36]. In the setting of allogeneic HSCT, CH is of particular interest because transplantation imposes a profound proliferative and immunologic stress on hematopoietic stem cells, creating a unique model to study clonal selection, donor-derived engraftment, recipient-derived persistence, and relapse-associated clonal evolution [37,38]. In this real-world cohort of recipients undergoing related donor HSCT, we show that post-transplant hematopoiesis is frequently sustained by donor-derived clones carrying CH-associated variants, particularly in *DNMT3A* and *TET2*. Several cases demonstrated stable transmission of donor variants to the recipient, whereas others showed progressive clearance of recipient-associated mutations after successful engraftment. These findings indicate that post-HSCT hematopoietic reconstitution extends beyond the conventional concept of donor-versus-recipient chimerism and instead reflects a dynamic molecular competition among donor-derived, recipient-derived, and newly emerging clones.

Allogeneic HSCT represents a profound clonal bottleneck [20]. Conditioning therapy, inflammatory remodeling of the marrow niche, immune pressure, and the proliferative demand required for hematopoietic recovery may favor the expansion of selected clones with increased fitness [3]. Our data support this concept: in patients with durable CR and full donor chimerism, recipient-associated mutations progressively disappeared and were replaced by donor-derived molecular signatures. Conversely, in patients with graft failure or relapse, recipient-associated oncogenic clones reappeared, or new disease-related clones emerged. Therefore, molecular reconstitution may precede or complement conventional chimerism analysis by providing qualitative information on the origin and biological behavior of post-transplant hematopoiesis [39]. The re-emergence of recipient-derived clones after HSCT may represent a molecular warning sign of graft failure or impending disease recurrence [40,41]. In UPN1, graft failure was accompanied by reappearance of the recipient *DNMT3A* oncogenic mutation, consistent with loss of donor hematopoiesis and restoration of recipient-derived CH. Similarly, in relapsed patients, post-transplant molecular evolution was characterized by increasing clonal complexity and, in selected cases, acquisition of new oncogenic alterations. Thus, longitudinal molecular profiling may complement, and in some cases precede, conventional chimerism assessment by providing qualitative insights into the clonal origin and biological behavior of post-transplant hematopoiesis [42].

*DNMT3A* was the most frequently altered gene in the entire cohort, involving donors and recipients before and after HSCT. This finding is consistent with the high prevalence of *DNMT3A* mutations in CH and with the known fitness advantage of *DNMT3A*-mutated hematopoietic stem cells [43,44]. However, the clinical meaning of *DNMT3A* mutations after HSCT depends on their origin and timing [45]. Donor-derived *DNMT3A* CH may stably contribute to hematopoietic reconstitution and has been associated in previous studies with reduced relapse risk, possibly through enhanced graft-versus-leukemia effects [46]. In contrast, persistence or reappearance of recipient-derived *DNMT3A* mutations may reflect residual disease, graft failure, or relapse-associated clonal hematopoiesis [47]. For this reason, *DNMT3A* mutations should not be uniformly interpreted as representing either benign clonal hematopoiesis or residual disease. Instead, their clinical significance should be interpreted within the context of donor genotype, chimerism status, disease status, and longitudinal changes in VAF.

A central potential clinically relevant finding of our study is the association between donor *TET2* Leu1721Trp and inferior recipient survival. All patients whose donors carried this variant died in our series, and OS was significantly shorter in this subgroup compared with recipients of WT donors or donors carrying other *TET2* variants. Importantly, the same variant was not associated with outcome when detected in recipients before or after HSCT, suggesting that its adverse impact may be context-dependent and could be linked to donor-derived hematopoietic reconstitution. The biological basis of this association remains merely speculative and hypothesis-generating, also because we lack an in vitro functional assay to prove the pathogenic role of this variant. *TET2*-mutated hematopoietic stem cells may display altered self-renewal, impaired differentiation, and enhanced inflammatory signaling [48]. In the post-transplant setting, the inflammatory marrow microenvironment generated by conditioning therapy and immune reconstitution could provide a selective advantage to specific *TET2*-mutated clones [49]. In this context, the donor-derived *TET2* Leu1721Trp variant may compromise the stability and efficiency of hematopoietic reconstitution, thereby promoting clonal remodeling, a pro-inflammatory milieu, and increased susceptibility to both disease relapse and non-relapse complications. However, because *TET2* is highly heterogeneous and variant-specific functional data are limited, these findings require cautious interpretation and external validation. The high VAF of this variant also suggests that *TET2* Leu1721Trp could represent a germline polymorphism rather than CH; however, it has been reported that several germline *TET2* variants, including Leu1721Trp, Pro363Leu, Val218Met, Leu34Phe, and His1778Arg, which may have also co-occurred, could represent a complex low-penetrant predisposing factors for myeloid neoplasm. Indeed, patients with TET2 SNPs are more likely to acquire a *TET2* somatic mutation vs. the WT form and vs. those subjects with somatic mutant carriers. Specifically, it has been reported that more than half of patients with myeloid neoplasms carrying a *TET2* variant had SNP Leu1721Trp in a biallelic fashion, suggesting a predisposition haplotype [50]. Although in accordance with previously published findings, our results are preliminary and only hypothesis-generating, because of a limited number of outcome events, very small subgroup sizes, and the lack of functional assay. In addition, we do not support indiscriminate exclusion of donors with CH, as most donor-derived variants, particularly VUS, were not associated with adverse outcomes and, in several patients, donor-derived CH supported stable hematopoietic reconstitution. Our data support a more refined approach based on variant type, gene involved, pathogenicity, VAF, donor age, recipient risk, and availability of alternative donors. In this framework, selected variants may identify donors or recipients who require closer molecular surveillance after HSCT.

We have already previously proposed both known pathogenic variants and VUS as a marker of CH, especially because certain VUS might represent germline polymorphisms predisposing to the acquisition of further somatic mutations [50,51,52]. When considering both pathogenic variants and VUS, CH is commonly found in AML, MDS, and CCUS patients, with high clonal complexity, especially in those subjects who will not respond to therapies [51]. Indeed, the phenomenon of CH is even more complex, as various clones can co-exist or sequential somatic mutations can accumulate throughout cell divisions, defining the condition of tumor heterogeneity characterized by the presence of neoplastic subclones, defined at the molecular and/or phenotypic levels, at different sites or within the same primitive lesion (spatial) or at different timepoints (temporal) [26,52,53]. Tumor heterogeneity within CH is the main driver of disease relapse, as a resistant clone can arise and replace the initiator [3]. In the setting of HSCT, during BM reconstitution, it has been described that recipients acquire an average of ~23 mutations, equivalent to ~1.5 years of aging, and *DNMT3A* and *TET2* are the most frequently mutated genes, reflecting the preferential growth of fitter clones under stress [3,54]. Moreover, higher rates of coalescent clones are documented at or after HSCT in recipient phylogenies [54,55,56,57,58], or in AML patients who relapse after transplantation [55]. In addition, fitter clones can show lineage bias and have poor responsiveness to proliferative stress, leading to ineffective hematopoiesis [58]. Therefore, clonal dynamics could represent an additional prognostic tool in hematological patients; however, CH and dynamics are almost exclusively studied at the molecular level by NGS or other highly expensive and time-consuming molecular biology assays [59]. In addition, currently, CH is defined only based on the presence of pathogenic or likely pathogenic mutations, while VUSs are not considered [60]. However, VUS might influence clinical outcomes, also because they might represent germline polymorphisms representing the seeding soil where leukemic clones can grow [3,50]. Here, we have added evidence to the possible synergistic pathogenic role of VUS in leukemia progression, also after HSCT, as they might represent a predisposing factor for the acquisition of further somatic mutations under proliferative stress, such as that during BM reconstitution after myeloablative regimens.

This study has limitations. The cohort was small, retrospective, and genetically heterogeneous, limiting subgroup analyses and preventing definitive conclusions regarding variant-specific prognostic effects. The 30-gene panel may have missed relapse-associated alterations outside the targeted regions, and functional validation of *TET2* Leu1721Trp was not performed. In addition, the limited number of patients carrying specific donor variants precludes a formal assessment of their independent prognostic value. Therefore, the association between the donor-derived *TET2* Leu1721Trp variant and adverse clinical outcome should be regarded as hypothesis-generating and requires validation in larger, independent cohorts. Despite these limitations, our study has major strengths, such as longitudinal molecular surveillance, paired donor–recipient sequencing, the use of a real-world clinical cohort, and the integration of molecular and clinical information. Moreover, we provide clinically relevant insights into post-transplant clonal dynamics. Integrating donor molecular profiling with serial NGS monitoring of recipients may improve the interpretation of post-HSCT hematopoietic reconstitution, facilitate discrimination between donor-derived clonal hematopoiesis and recipient-derived measurable residual disease (MRD), and enable the early identification of patients at increased risk of graft failure or disease relapse.

## 4. Materials and Methods

### 4.1. Patients

In this single-center observational retrospective study, clinical and molecular data were collected in an electronic database from a total of 20 allogeneic HSCT recipients and from their MR or haploidentical donors, followed at the Hematology and Transplant Center, University Hospital “San Giovanni di Dio e Ruggi d’Aragona”, Salerno, Italy, between October 2021 and February 2026. Inclusion criteria were as follows: age ≥ 18 years old; indications for allogeneic HSCT (e.g., MDS, AML, or acute lymphoblastic leukemia [ALL]); HLA-identical or haploidentical family donor availability; first allogeneic HSCT; and written informed consent. Exclusion criteria were as follows: age < 18 years old; ineligibility for allogeneic HSCT; lack of complete clinical data; missing molecular data; and lack of written informed consent. Diagnoses were made according to the 2016 and 2022 World Health Organization (WHO) criteria [27,28], and risk stratification was based on 2016 and 2022 European LeukemiaNet (ELN) system for AML and ALL patients [29,30], or the Revised International Prognostic Scoring System (IPSS-R) for MDS subjects [31]. Patients were treated according to international guidelines, and response to therapies was assessed according to the 2022 ELN response criteria, minimal measurable disease (MMR) status, and to the International Working Group (IWG) response criteria for MDS [29,30,32]. Peripheral blood stem cell donors were screened for hematological malignancies or for allogeneic HSCT eligibility according to the International Bone Marrow Donor Registry (IBMDR) guidelines [33]. Clinical characteristics of patients and donors are summarized in Table 2.

### 4.2. Molecular Profiling by NGS

For molecular profiling, peripheral blood (PB) and/or BM blood samples were collected as part of clinical practice: at diagnosis; at the end of induction therapy; before transplantation; during post-transplant follow-up for MMR monitoring, such as after three, six, 12, and 24 months post-HSCT; and at disease relapse. For each healthy donor, PB samples were obtained prior to the apheretic procedure for hematopoietic stem cell collection. Next, mononuclear cells were isolated by Ficoll–paque gradient centrifugation, and then subjected to genomic DNA extraction using a Maxwell CSC Blood DNA kit following the manufacturers’ instructions (Promega, Milan, Italy). NGS analysis was performed using SOPHIA Genetics Myeloid Solution panel (Sophia Genetics, Rolle, Switzerland), covering 30 genes (*ABL1*, *ASXL1*, *BRAF*, *CALR*, *CBL*, *CEBPA*, *CSF3R*, *DNMT3A*, *ETV6*, *EZH2*, *FLT3*, *HRAS*, *IDH1*, *IDH2*, *JAK2*, *KIT*, *KRAS*, *MPL*, *NPM1*, *NRAS*, *PTPN11*, *RUNX1*, *SETBP1*, *SF3B1*, *SRSF2*, *TET2*, *TP53*, *U2AF1*, *WT1*, *ZRSR2*), as previously described [26]. Briefly, samples were processed by first diluting with IDTE (10 mM Tris, 0.1 mM EDTA) to obtain 200 ng of DNA in 30 μL of final volume, and then enzymatic DNA fragmentation and barcode adapter ligation were carried out. Fragment purification (300–700 bp length) was performed using AMPure beads (Beckman Coulter, Milan, Italy); fragments were then amplified by PCR, and further quantified and quality checked using a 4200 Tape Station System (Agilent, Milan, Italy). For each sample, 150 ng of library was pooled, and 2 μL of universal blockers and 5 μL of human cot DNA were added. After lyophilization and DNA denaturation at 95 °C for 10 min, target-specific biotinylated probes were hybridized at 65 °C for 4 h, followed by streptavidin-coated bead addition, washing, and sample amplification by PCR. After a second purification step, quality and concentration of library was checked using a 4200 TapeStation System (Agilent), and dilution was carried out to obtain a final concentration of 4 nM. After library denaturation, 10 pM of denatured library and 5% of PhiX Control v3 Library (Illumina, Milan, Italy) were added to the flow cell for sequencing. Fastq files were loaded on SophiaDDM software (v.5.0; Sophia Genetics) for data alignment to the human genome GRCh37/hg19 with a minimum coverage of 500 reads, a reported sensitivity of 99.92%, and specificity of 99.99%. Non-intronic and non-synonymous modifications with a VAF > 5% were considered for diagnostic purposes, while for research purposes, those with a VAF > 0.5% were included in the analysis. Intronic variants and those in the UTR region were excluded. Variant pathogenicity was determined according to COSMIC and ClinVar databases, while variants not reported in these databases were classified as pathogenic based on in silico prediction tools [26].

### 4.3. Statistical Analysis

Data were collected in spreadsheets and analyzed using R software (v. 4.0.5; RStudio, Boston, MA, USA), SPSS (v. 25; IBM), and GraphPad Prism (v. 11.0, GraphPad software LLC, San Diego, CA, USA). Clinical, phenotypic, and molecular information was retrieved by reviewing medical records and laboratory and histopathological reports. In the present study, CH was defined as the presence of somatic variants in genes recurrently implicated in the pathogenesis of myeloid neoplasms, identified by NGS with VAF ≥ 5%, in the absence of sufficient morphological, cytogenetic, or clinical criteria for the diagnosis of active myeloid neoplasms, according to the 2022 WHO classification, and the 2022 International Consensus Classification (ICC) [34]. CHIP was defined by the presence of somatic mutations in AML-related genes with VAF ≥ 2%, in the absence of persistent cytopenias or other diagnostic evidence of hematologic malignancy [35]. Parametric and nonparametric tests were performed to compare continuous variables (*t*-test and Kruskal–Wallis test). Univariate logistic regression models were used to study the effects and related odds ratios of independent variables. Specifically, we included factors known to influence OS, such as donor and recipient sex [61], pre-HSCT status [62], and donor type [63]. *TET2* mutational status was added, based on the potential clinical relevance of *TET2* Leu1721Trp. In addition, the number of pre-transplant clones was included, as growing evidence is highlighting clonal heterogeneity as an independent prognostic factor in hematological patients after chemotherapy and/or HSCT [64,65]. Influence of confounding on clinical outcomes, such as disease type or donor/recipient age, was separately investigated by univariate analysis. Specifically, disease type (AML, *p* = 0.1904; ALL, *p* = 0.1142), donor age (*p* = 0.5776), type of conditioning regimen (*p* = 0.0579), or GvHD onset (*p* = 0.9235) were not associated with clinical outcomes. A *p* value < 0.05 was considered statistically significant. Clonal dynamics of patients before and after HSCT and donors are reported as balloon plots, made using reshape2 v.1.4.5, ggplot2 v.4.0.3, and ggpubr v.1.0.0 packages in RStudio software (v. 2025.09.0).

## 5. Conclusions

In conclusion, to date, donor CH/CHIP has been considered a low-risk or even protective phenomenon (in the case of *DNMT3A*), allowing the clinical use of donors with molecular alterations [66]. Current clinical practice tends to advise against routine donor screening due to the predominantly neutral nature of CHIP on OS and non-relapse-related mortality [67]; however, high-risk variants could affect post-transplant outcomes. Therefore, it is crucial to initiate large-scale multicenter studies to validate whether specific gene variants should become exclusion or intensive monitoring criteria. Indeed, donor-derived clones might interact differently with the recipient’s inflammatory microenvironment, thus driving or failing in the BM reconstitution, as CH in the context of allogeneic transplantation represents a complex biological phenomenon, under continuous proliferative stress [3]. Our findings do not support the indiscriminate exclusion of donors with CH, while they are exploratory and hypothesis-generating, supporting a future personalized approach based on molecular risk assessment and closer post-transplant surveillance when specific potentially high-risk variants are identified. Ultimately, the combination of donor molecular profiling with longitudinal monitoring of the recipient could refine donor–recipient matching, improve early detection of molecular relapse or transplant instability, and contribute to more personalized post-transplant management.

## Figures and Tables

**Table 1:** Multivariate analysis (dependent variable = overall survival).

Variable	Estimate	95%CI	|t|	*p* Value
Intercept	49.97	26.69 to 73.26	4.949	0.0011
Sex [F]	−12.77	−27.18 to 1.639	2.044	0.0752
Pre-transplant status [PR]	2.183	−35.49 to 39.86	0.134	0.8970
Pre-transplant status [CR]	21.11	−14.68 to 56.91	1.360	0.2108
Donor [MRD]	8.581	−6.751 to 23.91	1.291	0.2329
Donor sex [M]	−13.17	−33.61 to 7.263	1.486	0.1755
*TET2* donor status [WT]	19.21	−4.215 to 42.64	1.891	0.0953
*TET2* donor status [Leu1721Trp]	27.07	−4.406 to 58.54	1.983	0.0826
*TET2* recipient status [Leu1721Trp]	−11.22	−34.94 to 12.50	1.091	0.3070
*TET2* recipient status [WT]	−37.03	−61.31 to −12.76	3.518	0.0079
Number of pre-transplant clones	−4.224	−11.51 to 3.059	1.337	0.2179

Abbreviations. CR, complete remission; PR, partial remission; MRD, minimal residual disease; WT, wild type.

**Table 2:** Recipients’ and donors’ characteristics.

Characteristics	Recipients *N* = 20	Donors *N* = 20
Median age at transplant, years (range)	52 (45–60)	46 (17–66)
M/F, *n* (%)	11 (55)/9 (45)	8 (40)/12 (60)
Diagnosis, *n* (%)		-
AML	17 (85)	
MDS	2 (10)	
ALL	1 (5)	
2022 ELN risk category, *n* (%)		-
Favorable	5 (25)	
Intermediate	3 (15)	
Adverse	12 (60)	
Disease status at transplant, *n* (%)		-
CR	17 (85)	
PR	1 (5)	
MRD positivity	2 (10)	
Type of donor, *n* (%)		
Haploidentical	10 (50)	
Matched-related	10 (50)	
Sex matching, *n* (%)	12 (60)	
Type of conditioning regimen, *n* (%)		-
TBF	9 (45)	
BU-FLU	5 (25)	
RIC BU-FLU	3 (15)	
TREO-FLU	3 (15)	
GvHD, *n* (%)	10 (50)	-
Disease status after transplant, *n* (%)		-
Full donor chimerism	16 (80)	
Graft failure	4 (20)	
Median follow-up, months (range)	31.1 (4.9–58.1)	-
Status at last follow-up, *n* (%)		-
Alive	13 (65)	
Dead	7 (35)	

Abbreviations. AML, acute myeloid leukemia; MDS, myelodysplastic syndromes; ALL, acute lymphoblastic leukemia; ELN, European LeukemiaNet; CR, complete remission; PR, partial remission; MRD, minimal residual disease; TBF, thiotepa, busulfan, fludarabine; BU, busulfan; FLU, fludarabine; RIC, reduced intensity conditioning; TREO, treosulfan; GvHD, graft-versus-host disease.

## Data Availability

The data that support the findings of this study are available on request from the corresponding authors. The data are not publicly available due to privacy restrictions.

## Supplementary Materials

The following supporting information can be downloaded at https://www.mdpi.com/article/10.3390/ijms27188008/s1.

## Abbreviations

The following abbreviations are used in this manuscript:

CH	Clonal hematopoiesis
NGS	Next-generation sequencing
CHIP	Clonal hematopoiesis of indeterminate potential
AML	Acute myeloid leukemia
HSCT	Hematopoietic stem cell transplantation
BM	Bone marrow
MDS	Myelodysplastic syndromes
ALL	Acute lymphoblastic leukemia
HLA	Human leukocyte antigen
WHO	World Health Organization
ELN	European LeukemiaNet
R-IPSS	Revised International Prognostic Scoring System
MMR	Minimal measurable disease
IWG	International Working Group
IBMDR	International Bone Marrow Donor Registry
PB	Peripheral blood
VAF	Variant allele frequency
ICC	International Consensus Classification
VUS	Variant of uncertain significance
CR	Complete remission
OS	Overall survival
WT	Wild type
GvHD	Graft-versus-host disease
