# Supplementary material for: Clonal Dynamics After Allogeneic Hematopoietic Stem Cell Transplantation from Related Donors: A Case Series
Source: Int J Mol Sci. 2026 Sep 9;27(18):8008. doi: 10.3390/ijms27188008 (PMC13606576; doi:10.3390/ijms27188008)
Supplement: Supplementary file 1 [file ijms-27-08008-s001.zip › ijms-4495805-supplementary.pdf]

Supplementary Table S1. Molecular landscape of patients and matched-donors.

Abbreviations. UPN, unique patient number; HSCT, hematopoietic stem cell transplantation; MRD, matched-related donor; T1, +3 months; T2, +6 months; T3, +12 months; WT, wild type.

**UPN1**				
	DNMT3A	***KIT***	***SETBP1***	***TET2***
**Pre-HSCT**	Arg882His, 31.8%	WT	Val231Leu, 51.1%<br>Val1101Ile, 47.9%	Ile1762Val, 48.6%
**Haploidentical donor**	WT	Met541Leu, 99.1%	Val231Leu, 47.5%	Ile1762Val, 48.5%
**Graft failure T1**	Arg882His, 44.3%	WT	Val231Leu, 51.6% Val1101Ile, 48%	Ile1762Val, 51.9%
**Graft failure T2**	Arg882His, 38.8%	WT	Val231Leu, 51.2% Val1101Ile, 50.8%	Ile1762Val, 50.1%
**Graft failure T3**	Arg882His, 47%	WT	Val1101Ile, 47% Val231Leu, 50%	Ile1762Val, 51%

**UPN2**					
	CEBPA	***CSF3R***	***EZH2***	***ZRSR2***	***TET2***
**Diagnosis**	WT	Met231Thr, 49.7%<br>Glu346Arg, 50.1%	Asp185His, 48.7%	WT	Pro29Arg, 46.2%
**Post therapy T1**	WT	Gln346Arg, 52.4%<br>Met231Thr, 47.3%	Asp185His, 50.5%	WT	Pro29Arg, 46.8%
**Post therapy T2**	WT	Met231Thr, 47.4%<br>Gln346Arg, 46.7%	Asp185His, 49.7%	WT	Pro29Arg, 39.4%
**Post therapy T3**	WT	Gln346Arg, 50.4%<br>Met231Thr, 49.2%	Asp185His, 48.3%	WT	Pro29Arg, 49.9%
**Pre-HSCT**	WT	Met231Thr, 49.8%<br>Gln346Arg, 48.6%	Asp185His, 50%	WT	Pro29Arg, 48.2%
**Haploidentical donor**	WT	Met231Thr, 99.7%<br>Gln346Arg, 100%	Asp185His, 49.4%	WT	Pro29Arg, 50.2%
**Post-HSCT T1**	WT	WT	WT	Ser447_Arg448dup, 95%	WT
**Post-HSCT T2**	WT	WT	WT	Ser447_Arg448dup, 97.2%	WT
**Post-HSCT T3**	WT	WT	Asp185His, 48.3%	Ser447_Arg448dup, 97.4%	Pro29Arg, 50.5%
**Post-HSCT T4**	Pro189del, 6%	WT	Asp185His, 49.9%	Ser447_Arg448dup, 97.2%	Pro29Arg, 49.9%
**Post-HSCT T5**	Pro189del, 5%	WT	Asp185His, 50.4%	Ser447_Arg448dup, 97.8%	Pro29Arg, 50.2%
**Post-HSCT T6**	Pro189del, 4,3%	WT	Asp185His, 50.7%	Ser447_Arg448dup, 98.2%	Pro29Arg, 47.1%

UPN3					
	CEBPA	*DNMT3A*	*EZH2*	*SETBP1*	*TET2*
**Pre-HSCT**	WT	WT	WT	Val231Leu, 50%	Pro363Leu, 51%<br>Leu1721Trp, 50%
**Haploidentical donor**	WT	WT	Asp185His, 49.7%	Val231Leu, 48.5%<br>Val1101Ile, 46.7%	WT
**Post-HSCT T1**	WT	WT	Asp185His, 50.6%	Val231Leu, 48.3%<br>Val1101Ile, 52%	WT
**Post-HSCT T2**	Pro189del, 5%	WT	WT	WT	WT
**Post-HSCT T3**	Pro189del, 2,5%	Arg771, 2.78%	Asp185His, 47,3%	Val231Leu, 47,9%<br>Val1101Ile, 45,2%	WT

UPN5						
	ASXL1	*CEBPA*	*CSF3R*	*MPL*	*TP53*	*TET2*
**Diagnosis**	WT	WT	WT	WT	WT	Arg283Cys, 47.5%<br>Ile1762Val, 50.7%
**Pre-HSCT**	WT	WT	WT	WT	Arg283Cys, 48%	Ile1762Val, 47%
**MRD donor**	Leu815Pro, 99.9%	His195_Pro196dup, 48.2%	Glu149Asp, 49.7%	WT	WT	WT
**Post-HSCT T1**	WT	His195_Pro196dup, 43.5%	Glu149Asp, 45.8%	WT	Arg283Cys, 1.3%	Ile1762Val, 1.7%
**Post-HSCT T2**	WT	His195_Pro196dup, 44.1%	Glu149Asp, 47.5%	WT	Arg283Cys, 1%	Ile1762Val, 1.1%
**Post-HSCT T3**	WT	His195_Pro196dup, 42.2%	Glu149Asp, 50%	Leu513del, 5%	WT	WT

UPN6				
	ASXL1	*EZH2*	*SETBP1*	*TET2*
**Pre-HSCT**	WT	Asp185His, 48.1%	Pro1130Thr, 46.9%<br>Val1101Ile, 52.2%	Leu1721Trp, 49.4%<br>Ile1762Val, 50.8%
**Haploidentical donor**	WT	WT	Pro1130Thr, 46.3%<br>Val231Leu, 47.4%	Ile1762Val, 53.5%
**Post-HSCT T1**	WT	Asp185His, 34%	Pro1130Thr, 51%<br>Val1101Ile, 32%<br>Val231Leu, 16%	Leu1721Trp, 33%<br>Ile1762Val, 51%
**Post-HSCT T2**	WT	Asp185His, 47.5%	Val1101Ile, 48.8%	WT
**Post-HSCT T3**	Gly645Valfs*58, 1.9%	WT	WT	Leu1721Trp, 48.9%

**UPN7**			
	CEBPA	***DNMT3A***	***TET2***
**Post-HSCT T1**	Pro189del, 5%	Met864Argfs*6, 8.5%<br>Cys514Alafs*137, 5.6%	Pro29Arg, 49.2%
**Post-HSCT T2**	Pro189del, 6.8%	Met864Argfs*6, 6.7%<br>Cys514Alafs*137, 12.1%	Pro29Arg, 48.8%<br>Leu1721Trp, 47.5%
**MRD donor**	Pro189del, 5.1%	Met864Argfs*6, 4.5%<br>Cys514Alafs*137, 3.5%	Pro29Arg, 52%<br>Leu1721Trp, 51%

**UPN8**						
	CSF3R	***EZH2***	***JAK2***	***NRAS***	***RUNX1***	***TET2***
**Diagnosis**	WT	WT	Pro97Ser, 44%	Gly13Arg, 12%<br>Gly13Asp, 7.3%	Lys110Gln, 45%	Leu1721Trp, 99%<br>Pro363Leu, 48%
**Post induction**	WT	WT	Pro97Ser, 49.3%	Gly13Arg, 9.7%<br>Gly13Asp, 15.5%	Lys110Gln, 60.9%	Leu1721Trp, 49.3%<br>Pro363Leu, 47.9%
**Post consolidation**	WT	WT	Pro97Ser, 48.2%	WT	WT	Pro363Leu, 50.4%<br>Leu1721Trp, 98%
**Pre-HSCT**	WT	WT	Pro97Ser, 47%	WT	WT	Leu1721Trp, 100%<br>Pro363Leu, 48%
**Haploidentical donor**	Glu808Lys, 49.8%	WT	Pro97Ser, 47.3%	WT	WT	Leu1721Trp, 46.7%
**Post-HSCT T1**	Glu808Lys, 47.8%	WT	Pro97Ser, 50.8%	WT	WT	WT
**Post-HSCT T2**	Glu808Lys, 48.9%	Glu404del, 2.4%	Pro97Ser, 48.2%	WT	WT	Leu1721Trp, 48.9%<br>Gln1548del, 3.2%
**Relapse**	WT	WT	Pro97Ser, 46.8%	Gln61Lys, 13.5%	Lys83Gln, 33.5%	WT

**UPN9**					
	DNMT3A	***IDH1***	***NPM1***	***NRAS***	***TET2***
**Diagnosis**	Cys559Phe, 44.8%	Arg132His, 45.1%	Trp288Cysfs*?, 45.2%	Gly13Asp, 34.9%	WT
**Post therapy**	WT	WT	WT	WT	WT
**Pre-HSCT**	WT	WT	WT	WT	WT
**MRD donor**	WT	WT	WT	WT	WT
**Post-HSCT T1**	WT	WT	WT	WT	Ile1762Val, 100%

UPN10							
	CEBPA	***CSF3R***	***DNMT3A***	***JAK2***	***NPM1***	***NRAS***	***TET2***
**Diagnosis**	WT	Thr476Ser, 53%	Arg736Pro, 47%	Arg564Leu, 48.1%	Trp288Cysfs*?, 44.9%	Gln61Arg, 46.1%	WT
**Post therapy T1**	Pro189del, 5%	Thr476Ser, 52%	Arg736Pro, 5.9%	Arg564Leu, 52%	WT	WT	WT
**Post therapy T2**	Pro189del, 5.1%	Thr476Ser, 51.3%	Arg736Pro, 11.6%	Arg564Leu, 50.8%	WT	WT	WT
**Pre-HSCT**	Pro189del, 5.5%	Thr476Ser, 47.4%	Arg736Pro, 7.3%	Arg564Leu, 50.1%	WT	WT	WT
**MRD donor**	WT	Asp510His, 50%	WT	WT	WT	WT	WT
**Post-HSCT T1**	Pro189del, 3.3%	Asp510His, 45.5%	WT	WT	WT	WT	Ser1518*, 0.97%

UPN11						
	CEBPA	***CSF3R***	***EZH2***	***NPM1***	***PTPN11***	***TET2***
**Diagnosis**	WT	Arg440Gln, 50.3%	WT	Trp288Cysfs*?, 47.4%	Ala72Gly, 43.7%	Pro363Leu, 50.3%<br>Leu1721Trp, 50%
**Pre-HSCT**	WT	Arg440Gln, 50.5%	WT	WT	WT	Pro363Leu, 50.6%<br>Leu1721Trp, 49.3%
**MRD donor**	WT	WT	Glu404del, 1.1%	WT	WT	Gln1548del, 1.6%
**Post-HSCT T1**	Pro189del, 5.1%	Arg440Gln, 50%	WT	WT	WT	WT
**Post-HSCT T2**	Pro189del, 5.1%	WT	WT	WT	WT	WT
**Post-HSCT T3**	c,2409-2A>G, 5.1%	Arg440Gln, 45.7%	WT	WT	WT	Gln1548del, 0.6%

UPN12			
	CEBPA	***TP53***	***TET2***
**Diagnosis**	His195_Pro196dup, 48%	Lys164Glu, 12%	WT
**Post induction**	His195_Pro196dup, 44%	WT	WT
**Pre-HSCT**	His195_Pro196dup, 44.3%	WT	WT
**Haploidentical donor**	WT	WT	Leu1721Trp, 49.1%<br>Pro363Leu, 49.2%

UPN13								
	ABL-1	***CEBPA***	***EZH2***	***FLT3***	***KIT***	***RUNX1***	***U2AF1***	***TET2***
**Diagnosis**	WT	Asn281Glufs*38, 17.9%	WT	Glu611_Phe612ins21, 35.6%<br>Glu604_Phe605ins16, 5.8%<br>Arg607_Glu608ins12, 8.5%	WT	Gly165Cys, 18.5%	Ser34Phe, 50.1%	Pro29Arg, 54.3%
**Post induction**	WT	Asn281Glufs*38, 17.9%	WT	WT	WT	WT	Ser34Phe, 17.9%	Pro29Arg, 49.9%
**Pre-HSCT**	WT	Pro189del, 7.2%	Glu404del, 1.8%	WT	WT	WT	Ser34Phe, 5.5%	Gln1548del, 1.6%<br>Pro29Arg, 49.7%
**MRD donor**	WT	WT	WT	WT	WT	WT	WT	Pro29Arg, 50.6%
**Post HSCT T1**	Val304Ile, 46.5%	WT	WT	WT	Val48Ile, 4.6%	Gly165Cys, 1.1%	Ser34Phe, 1.9%	WT
**Post HSCT T2**	WT	WT	WT	WT	WT	WT	WT	WT

UPN14								
	ASXL1	***CBL***	***CEBPA***	***DNMT3A***	***MPL***	***RUNX1***	***SETBP1***	***TET2***
**Diagnosis**	Gly646Trpfs*12, 38.5%	Lys382Arg, 42.5%	WT	WT	WT	Gly165Ser, 43.3%	Val1101Ile, 50.2%	Val1718Leu, 49%<br>Gly355Asp, 49.4%
**Post induction**	Gly646Trpfs*12, 12.9%	.Lys382Arg, 13.9%	WT	WT	WT	Gly165Ser, 1%	Val1101Ile, 47.9%	Val1718Leu, 47.9%<br>Gly355Asp, 49.8%
**Post consolidation**	Gly646Trpfs*12, 17.3%	WT	Pro189del, 5.4%	WT	WT	WT	WT	WT
**Pre-HSCT**	Gly646Trpfs*12, 24.7%	Lys382Arg, 27.1%	Pro189del, 4.7%	WT	WT	Gly165Ser, 12.2%	WT	WT
**MRD donor**	WT	WT	WT	WT	Leu513de l, 2.3%	WT	WT	Gly355Asp, 46.6%
**Post HSCT T1**	Gly646Trpfs*12, 20%	WT	WT	WT	WT	WT	WT	WT
**Post HSCT T2**	WT	WT	WT	Arg891Profs*30, 1.24%	WT	WT	WT	WT
**Post HSCT T3**	WT	WT	WT	Arg891Profs*30, 1.09%<br>Ile310Leufs*6, 5,42	WT	WT	Val1101Ile, 44.1%	Glu1162Aspfs*63, 1.4%<br>Ile1762Val 45.2%<br>Val1718Leu 44.1%<br>Gly355Asp 49.5

**UPN15**						
	BRAF	***DNMT3A***	***EZH2***	***IDH2***	***NRAS***	***TET2***
**Diagnosis**	Val600Glu, 5.8%	Arg882His, 15.8%	WT	Arg140Gln, 17.3%	WT	WT
**Post induction**	WT	Arg882His, 29.4%	WT	Arg140Gln, 31%	Gln61Pro, 11.5%	WT
**Pre-HSCT**	WT	Arg882His, 22.8%	WT	Arg140Gln, 23.5%	Gln61Pro, 9.4%	WT
**Haploidentical donor**	WT	WT	WT	WT	WT	WT
**Post HSCT T1**	WT	WT	Asp189del, 1%	WT	WT	WT
**Post HSCT T2**	WT	WT	WT	WT	WT	WT
**Post HSCT T3**	WT	Ile840Serfs*8, 14.26%	WT	WT	WT	Lys655Asnfs*45, 0.8%<br>Ile1762 46.3%

**UPN16**					
	CEBPA	***DNMT3A***	***EZH2***	***IDH1***	***ZRSR2***
**Pre-HSCT**	WT	Ser663*, 7.4%	WT	Arg132Cys, 6.6%	Arg448dup, 50.3%
**MRD donor**	His195Pro, 1.4%	WT	Asp185His, 48.6%	WT	WT
**Post HSCT T1**	WT	Val441Trpfs*210, 5.2%	WT	WT	Arg448dup, 50.8%
**Post HSCT T2**	WT	Val441Trpfs*210, 5.2%	WT	WT	WT
**Post HSCT T3**	WT	Val441Trpfs*210, 6.73%	WT	WT	WT

**UPN17**				
	ABL-1	***DNMT3A***	***MPL***	***RUNX1***
**Diagnosis**	Phe359Cys, 39.4%	ex.23, p.?, 41.3%	Leu513del, 2.5%	ex.5, p.?, 40%
**Pre-HSCT**	WT	ex.23, p.?, 2.5%	WT	ex.5, p.?, 0.5%
**Haploidentical donor**	WT	WT	WT	WT

**UPN18**							
	CBL	***DNMT3A***	***EZH2***	***JAK2***	***KIT***	***NRAS***	***TET2***
**Diagnosis**	ex.8,p.?, 7.5%	WT	Arg690His, 45.3%	Gly571Ser, 50.9%	Asp816Val, 2.6%	Gly13Asp, 26.9%	WT
**Post induction**	WT	Asp531del, 3.5%	WT	Gly571Ser, 47.5%	WT	WT	His1778Arg, 47.5%
**Pre-HSCT**	WT	WT	WT	Gly571Ser, 49.7%	WT	WT	WT
**Haploidentical donor**	WT	WT	WT	WT	WT	WT	WT

**UPN20**					
	CEBPA	***DNMT3A***	***FLT3***	***NPM1***	***TET2***
**Diagnosis**	Arg343Alafs*79, 46.5%	Tyr735Cys, 41.4%	Arg595—Glu596ins12, 31.1%	pTrp288Cysfs*, 35.7%	WT
**Pre-HSCT**	WT	WT	WT	WT	Ile1762Val, 47.5%<br>Gly355Asp, 49.5%
**Haploidentical donor**	WT	WT	WT	WT	Pro29Arg, 47.4%
**Post HSCT T1**	WT	WT	WT	WT	Pro29Arg, 49.3%
